# AI-Based Estimation of End-Systolic Elastance From Arm-Pressure and Systolic Time Intervals

**DOI:** 10.3389/frai.2021.579541

**Published:** 2021-04-14

**Authors:** Vasiliki Bikia, Dionysios Adamopoulos, Stamatia Pagoulatou, Georgios Rovas, Nikolaos Stergiopulos

**Affiliations:** ^1^ Laboratory of Hemodynamics and Cardiovascular Technology, Institute of Bioengineering, Swiss Federal Institute of Technology, Lausanne, Switzerland; ^2^ Cardiology Department, Geneva University Hospitals, Geneva, Switzerland

**Keywords:** cardiac monitoring, contractility, heart, noninvasive, regression analysis

## Abstract

Left ventricular end-systolic elastance (E_es_) is a major determinant of cardiac systolic function and ventricular-arterial interaction. Previous methods for the E_es_ estimation require the use of the echocardiographic ejection fraction (EF). However, given that EF expresses the stroke volume as a fraction of end-diastolic volume (EDV), accurate interpretation of EF is attainable only with the additional measurement of EDV. Hence, there is still need for a simple, reliable, noninvasive method to estimate E_es_. This study proposes a novel artificial intelligence—based approach to estimate E_es_ using the information embedded in clinically relevant systolic time intervals, namely the pre-ejection period (PEP) and ejection time (ET). We developed a training/testing scheme using virtual subjects (*n* = 4,645) from a previously validated in-silico model. Extreme Gradient Boosting regressor was employed to model E_es_ using as inputs arm cuff pressure, PEP, and ET. Results showed that E_es_ can be predicted with high accuracy achieving a normalized RMSE equal to 9.15% (r = 0.92) for a wide range of E_es_ values from 1.2 to 4.5 mmHg/ml. The proposed model was found to be less sensitive to measurement errors (±10–30% of the actual value) in blood pressure, presenting low test errors for the different levels of noise (RMSE did not exceed 0.32 mmHg/ml). In contrast, a high sensitivity was reported for measurements errors in the systolic timing features. It was demonstrated that E_es_ can be reliably estimated from the traditional arm-pressure and echocardiographic PEP and ET. This approach constitutes a step towards the development of an easy and clinically applicable method for assessing left ventricular systolic function.

## Introduction

The concept of end-systolic elastance (E_es_), first introduced by Suga *et al.* (Suga and Sagawa, 1974), has become widely accepted. The E_es_, i.e., the slope of the end-systolic pressure-volume relationship (ESPVR), constitutes a pivotal determinant of left ventricular (LV) systolic performance and is now considered an established index of contractility (Suga et al., 1973; Suga and Sagawa, 1974; Sagawa et al., 1977). Assessment of E_es_ is of high importance in physiological studies and clinical practice. The effective matching between E_es_ and vascular load leads to optimal mechanical function. Age-related arterial stiffening (Chen et al., 1998) and hypertension (Borlaug et al., 2009) are related to the stiffening of the left ventricle, which is accompanied by an increased value of E_es_. It has also been shown that antihypertensive treatment reduces E_es_ and enhances arterial-ventricular coupling (Lam et al., 2013). Furthermore, the intercept of the ESPVR has been linked with prognosis in chronic heart failure (Ky et al., 2013). Derivation of E_es_ requires the measurement of multiple invasive pressure-volume (P-V) loops under various loading conditions which limits its use in the routine clinical setting. In an attempt to address this limitation, research has been directed towards the development of methods for deriving E_es_ from easily obtained noninvasive single-beat measurements (Shishido et al., 2000; Chen et al., 2001; Pagoulatou et al., 2021).

In our previous work (Bikia et al., 2020), we demonstrated that E_es_ could be accurately determined using brachial systolic (brSBP) and diastolic blood pressure (brDBP), heart rate (HR), and ejection fraction (EF). The importance of EF on obtaining an accurate E_es_ estimation has been also indicated by other published methods (Shishido et al., 2000; Chen et al., 2001). Nevertheless, accurate interpretation of EF renders essential the additional knowledge of physical determinants of myocardial contraction, namely, the preload and afterload (Krayenbühl et al., 1968; Konstam and Abboud, 2017). The question that arises is whether E_es_ could be derived in a faster and more optimized way while reducing the complexity of the required measurements. Our primary hypothesis is that EF information could be replaced by other cardiac functional parameters, e.g., electrical or acoustic signals of cardiac events, that are related to the LV contractility in a direct or indirect manner.

Previous studies have highlighted the relevance of the timing of cardiac events in assessing the contractile state of the heart (Weissler et al., 1968; Weissler et al., 1981; Boudoulas, 1990). Pre-ejection period (PEP), i.e., the period between the onset of ventricular contraction and the aortic valve opening, serves as a major index of excitation-contraction coupling and may potentially be used to evaluate contractility (Gillebert et al., 2004; Krohova et al., 2017). Concurrently, LV ejection time (ET), delimited by the opening and closing of the aortic valve, provides incremental prognostic information on cardiac performance (Boudoulas, 1990; Biering-Sørensen et al., 2018).

The objective of this study was to propose a novel method for the estimation of E_es_ using brSBP, brDBP, HR (via sphygmomanometry), and contractility-related timing parameters (via ECG and echocardiography), i.e., PEP and ET. The analysis relied on the use of Machine Learning regression analysis. To appraise our concept, we developed and evaluated this method using synthetic data generated from a previously validated in-silico model (Reymond et al., 2009). An in-silico model constitutes a computer program that allows for simulating human physiology, cardiovascular mechanisms, and/or progression of disease. The utility of such models in medicine has essentially facilitated the visualization and prediction of physiological responses under different cardiovascular conditions. In the present study, the in-silico model provides additional hemodynamic insights, which would be difficult to acquire *in vivo*, and is used for the preliminary assessment and design of the proposed methodology.

## Materials and Methods

### Data Analysis

#### Study Population

The population used in the present in-silico study reflected a wide range of hemodynamical properties and states. Different hemodynamic cases (*n* = 4,645) were simulated by modifying key cardiac and systemic parameters of a previously validated in-silico model. The 1-D mathematical cardiovascular model, which was adopted in the current study, has been well described in (Reymond et al., 2009). The arterial tree model incorporates all the major arteries of the systemic circulation, as well as a detailed network representation of the cerebral circulation and the coronary circulation. The governing equations of the model are acquired by integrating the longitudinal momentum and continuity of the Navier-Stokes equations over the arterial cross-sectional area. By solving the governing equations with proper boundary conditions, flow and pressure are obtained in all arterial locations. The arterial segments of the model are considered as long tapered tubes, and their compliance is calculated by a nonlinear function of pressure and location as described by Langewouters (Langewouters, 1982). Distal vessels are terminated with three-element Windkessel models (Westerhof et al., 2009) and intimal shear is modeled using the Witzig-Womersley theory (Womersley, 1957). At the proximal end, the arterial tree is coupled with a varying elastance model of the left ventricle (Suga and Sagawa, 1974; Sagawa et al., 1977). This time-varying elastance model (VEM) describes the relationship between the LV pressure, P_LV_, and volume, V_LV_, namely:

$$E\left(t\right)=\frac{P_{\mathrm{LV}}\left(t\right)}{V_{\mathrm{LV}}\left(t\right)-V_{d}},$$ (1)

where V_d_ indicates the dead volume of the left ventricle. Further details on the 1-D model can be found in the original publications (Reymond et al., 2009; Reymond et al., 2011).

Concerning data generation, E_es_ varied in the range of 1.00–4.50 mmHg/ml so that the dataset includes cases with normal as well as dilated and hypertrophied hearts (Feldman et al., 1996; Chen et al., 1998; Pak et al., 1998). The filling pressure lied in the range of 7.00–23.00 mmHg according to (Feldman et al., 1996; Chen et al., 1998; Pak et al., 1998). The dead volume (V_d_) and the time of maximal elastance (t_es_) were modified according to (Starling et al., 1987; Reymond et al., 2009). HR values were within the range of 60 and 100 bpm. Total peripheral resistance and arterial compliance were altered to simulate a wide variety of arterial tree configurations (Langewouters, 1982; Lu and Mukkamala, 2006; Segers et al., 2008). In addition to the modification of cardiac and systemic parameters, arterial geometry was changed with respect to arterial length and diameter for each segment to approximate different body types (Wolak et al., 2008; Devereux et al., 2012). The variation of the geometry was done in a uniform way for all arterial segments based on the variation of the aortic diameter. No topological variations (e.g., in the circle of Willis, number of branches from aortic arch, etc.) were considered. Nonuniform aortic stiffening was considered for the elderly and hypertensive virtual subjects following the approach described in Bikia *et al.* (Bikia et al., 2019).

Given that the literature data are only provided in terms of mean and standard deviation or/and minimum and maximum values, we chose to perform random Gaussian sampling for varying the model’s parameters. We filtered the generated data to ensure that they correspond to physiological human conditions. Concretely, the physiological validity of each subject was assessed by comparing the simulated brachial and aortic systolic blood pressure (SBP), DBP, MAP, and pulse pressure (PP) to the reference values reported in the previous studies by McEniery (McEniery et al., 2005) (normotensive cases) and Bordin Pelazza and Filho (Pelazza and Filho, 2017) (hypertensive cases). A subject was discarded from the dataset if any of the blood pressure values was not satisfying the minimum and maximum thresholds indicated as mean ± 2.807SD (99.5% confidence intervals). Such an approach for generating synthetic data has been applied by a previous similar study (Charlton et al., 2019).

### Features Extraction

The relevant features were extracted from the flow and pressure waves produced by the in-silico model. Synthetic brSBP, brDBP, brPP as well as HR data were calculated from the pressure wave at the left brachial artery.

**FIGURE 1:**
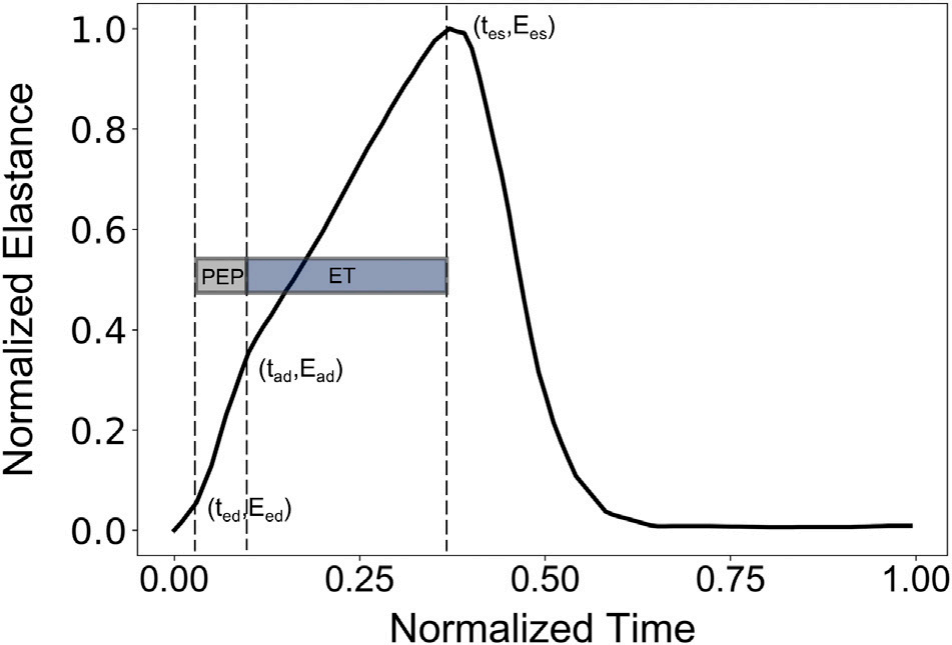
Representative elastance curve E(t) with the indicated t_ed_ (early time point of isovolumic contraction), t_ad_ (ending time point of isovolumic contraction), and t_es_ (end-systolic time point).

Normally, PEP and ET could be extracted from the synchronous recordings of the aortic blood flow and the ECG signal. Here, the values of PEP and ET were derived following Shishido et al. (Shishido et al., 2000), as illustrated in Figure 1. The reason that we employed this approach to calculate PEP and ET was the absence of a model of cardiac electrical activity that would indicate the starting position of Q-wave. PEP was calculated as the duration of the isovolumic contraction. The early isovolumic point (t_ed_) was defined as the time point when the time derivative of LV pressure is above 30% of dP/dt_max_. The end of the isovolumic contraction (t_ad_) was calculated from the first inflection point of the elastance curve at the upstroke area. End-systole (t_es_) was measured as the time point when dP/dt reaches 20% of dP/dt_min_. PEP and ET were obtained as t_ad_-t_ed_ and t_es_-t_ad_, respectively.

### Regression Analysis

The dataset was organized in pairs of inputs and outputs in order to be used for the training/testing process. The input features included the “measured” brSBP, brDBP, HR, PEP, and ET, as well as the t_ed_, t_ad_, and t_es_. The inclusion of the latter timing points was done to improve the descriptive cardiovascular profile of each subject and further enhance the regressor’s performance. Furthermore, a predictive model was developed including stroke volume (SV) and ejection fraction (EF) as additional input features. Hence, three predictive models were developed and evaluated based on the different inputs’ sets: i) one using brSBP, brDBP, HR, PEP, ET, t_ed_, t_ad_, and t_es_ (M1), ii) a second one with only brSBP, brDBP, HR, PEP, and ET (M2), and finally, iii) a third model including all features from model M1 as well as SV and EF (M3). We additionally investigated the predictive capacity of our framework to estimate V_d_. Nevertheless, the estimation of V_d_ was not considered as the main focus of the present study.

We used Extreme Gradient Boosting (XGB) (Chen and Guestrin, 2016) for the regression analysis. The 70% of the dataset (3,251 subjects) was used for the training of the XGB model. The remaining 30% (1,394 subjects) was kept for the testing. The regressor f(.) was described as $Y_{E_{es}}\approx \;f_{E_{es}}\left(X;\beta \right)$, where *β* represents the unknown model parameters, X, the independent variables, and Y_Ees,_ the dependent variable. The unknown parameters of the model were optimized via an inner cross validation loop, i.e., hyperparameter tuning. Hyperparameter tuning was performed using GridSearch with 10-fold cross validation. The hyperparameters that were chosen to be optimized are reported in Table 1 below. The hyperparameters’ values that are not reported in Table 1 were set to their default value. The selected hyperparameters’ values for the six predictive models are also reported in Table 2. Consequently, the prediction accuracy for each regression model was evaluated on a subject level.

**TABLE 1:** List of the hyperparameters which were chosen to be optimized and their corresponding values.

Hyperparameter	Values
*learning_rate*	{0.005, 0.01, 0.05, 0.1, 0.15}
*max_depth*	{3, 5, 10}
*n_estimators*	{500, 750, 1,000, 1,250, 1,500, 1750}

**TABLE 2:** List of the selected hyperparameters for all the predictive models.

Model	Selected hyperparameters		
	*learning_rate*	*max_depth*	*n_estimators*
XGB_Ees_ M1	0.05	3	1,750
XGB_Ees_ M2	0.01	3	1,500
XGB_Ees_ M3	0.1	3	1,250
XGB_Vd_ M1	0.01	3	500
XGB_Vd_ M2	0.01	3	500
XGB_Vd_ M3	0.1	3	1,750

We assessed the importance of each input feature using two concepts, i.e., the feature importance scores returned by the XGB model, and the permutation feature importances. A major difference between the two concepts is that the feature importances from XGB are calculated based on the learning process through the training data, while the permutation feature importances are yielded from the estimations on a test set.

More specifically, the feature importance by XGB provides a score that indicates how useful and valuable each feature was in the construction of the boosted decision trees within the model. The hierarchical structure of a decision tree leads us to the final prediction by traversing through the nodes of the tree. Each node consists of a feature which is further split into more nodes as the tree develops vertically. The more times a feature is used to make key decisions with decision trees, the higher its relative importance. Formally, the feature importance score is calculated for a single decision tree by the amount that each feature split point improves the performance measure, weighted by the number of observations the node is responsible for. The feature importance scores are then averaged across all of the trees within the model. This importance is calculated explicitly for each feature and allows features to be ranked and compared to each other.

We additionally provide the permutation feature importances which are helpful to interpret the changes in model’s performance when the information of a feature is discarded. The concept of permutation feature importances relies on measuring the importance of a feature by calculating the increase in the model’s prediction error after permuting the feature. Permutation of a feature is achieved by shuffling the values of the feature on the test set. A feature is considered as significant if shuffling its values increases the (trained) model error, demonstrating that the model relied on the feature for the prediction. A feature is unimportant if shuffling its values does not change the model error, showing that the model ignored the feature for the prediction. The concept of permutation feature importance was first introduced by Breiman (Breiman, 2001). Essentially, permutation feature importances express the increase in model error when the feature’s information is destroyed. For calculating the permutation importances, we randomly shuffled the values of each feature and we computed the RMSE after the permutation. This was repeated 20 times and the mean and standard deviation of the increase in RMSE were reported.

**FIGURE 2:**
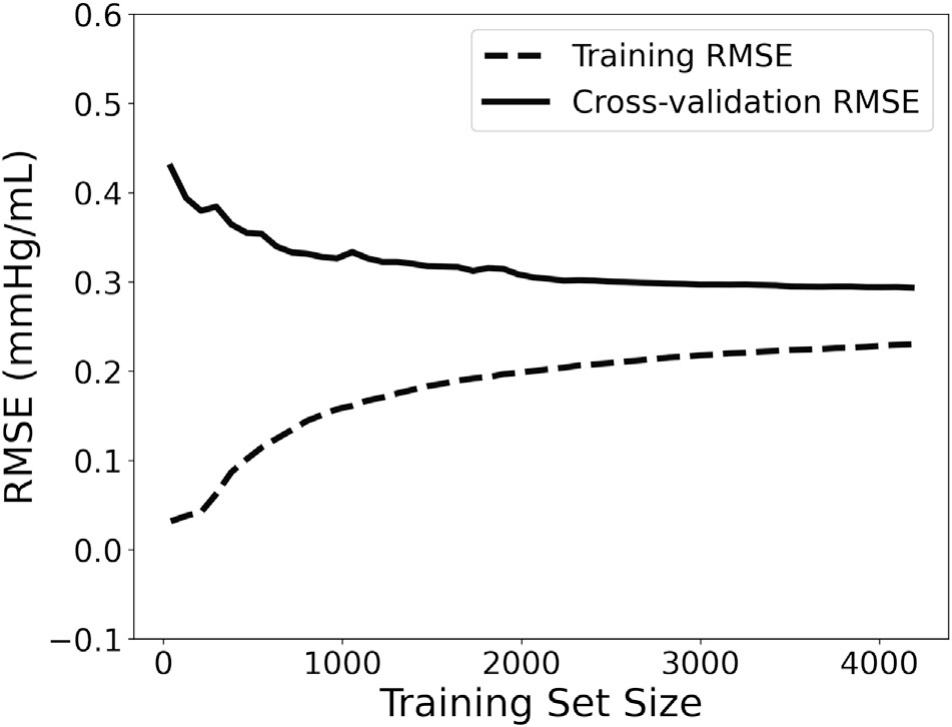
Learning curve visualizing the effect of the number of data instances on the performance. RMSE: root mean squared error.

Moreover, the accuracy of a Machine Learning regressor is largely dependent on the size of the initial training datasets. Thus, the investigation of how large a training dataset needs to be in order to build a reliable predictive model is imperative. To obtain this information the learning curve was computed. Learning curves allow for visualizing the effect of the number of data instances on the performance. The learning curve was fitted using the observed accuracy (in terms of RMSE) according to a given training sample size. The training size was modified from 1 to 98% of the total number of subjects (50 samples of training size). The learning curve is presented in Figure 2. We observed that as the number of training data increases, the RMSE of testing decreases and starts saturating while approaching the 4,000 data instances. Given that it is not clear whether a steady state is utterly achieved (a state where no substantial improvement occurs by increasing the number of training data), we decided to include all the training dataset for performing the regression analysis. Hence, the model with the selected hyperparameters was fit to the entire training set (*n* = 3,251), and the performance metrics reported in the Results’ section correspond to the testing set (*n* = 1,394). The training/testing pipeline was implemented using the Scikit-learn library (Pedregosa et al., 2011) in a Python programming environment. The pandas and NumPy packages were also used (Oliphant, 2006; McKinney, 2010).

### Sensitivity to Noise

We assessed the sensitivity of our model to errors in the measurement of PEP and ET. In addition, sensitivity analysis was performed for errors in the blood pressure measurements (i.e., amplitude of brachial pressure waveform). The data were artificially corrupted using three levels of errors, i.e., ±10%, ±20%, and ±30% with respect to their actual value. Errors in measurements were simulated with a random distribution, i.e., for a noise level equal to ±20%, the error of each measurement was randomly drawn from the range of [-20, 20] %. The effect of erroneous inputs was evaluated and the model’s performance was reported for the six experiments [3 noise levels x 2 sets of inputs (systolic timing intervals and blood pressure values)]. The experiments were performed using the hyperparameters which were selected from the M1 model (Table 2) which did not account for the noise.

### Statistical Analysis

The statistical analysis was performed in Python (Python Software Foundation, Python Language Reference, version 3.6.8, Available at http://www.python.org). All values are presented as mean ± SD. The agreement, bias, and precision between the model predictions and the real values were evaluated by using the Pearson’s correlation coefficient (r), the mean absolute error (MAE), the normalized root mean square error (nRMSE), and the Bland-Altman analysis (Bland and Altman, 1986). The computed nRMSE was based on the difference between the minimum and maximum values of the dependent variable (y) and was computed as RMSE/(y_max_ – y_min_). Linear least-squares regression was performed for the estimated and reference data. The slope and the intercept of the regression line were reported. Two-sided *p*-value for a hypothesis test whose null hypothesis is that the slope is zero, using Wald Test with *t*-distribution of the test statistic, was calculated. A *p*-value below 0.05 was considered as statistically significant.

## Results

Table 3 summarizes the cardiac and vascular characteristics of the 4,645 subjects included in this study.

**TABLE 3:** Summary of the cardiovascular characteristics of the virtual study cohort (*n* = 4,645).

Variable	mean ± SD *n* = 4,645
End-systolic elastance [mmHg/ml]	3.06 ± 0.74
End-diastolic elastance [mmHg/ml]	0.13 ± 0.04
Filling pressure [mmHg]	15.32 ± 3.47
Heart rate [bpm]	79.61 ± 8.27
Dead volume [ml]	22.68 ± 14.07
Ejection fraction [%]	53.74 ± 9.33
t_es_ [ms]	355.09 ± 26.24
t_ad_ [ms]	65.75 ± 18.46
t_ed_ [ms]	13.25 ± 1.02
Pre-ejection time [ms]	52.5 ± 18.19
Ejection time [ms]	289.35 ± 26.85
Stroke volume [ml]	78.7 ± 21.62
Aortic SBP [mmHg]	132.32 ± 24.67
Aortic DBP [mmHg]	100.73 ± 16.97
Aortic PP [mmHg]	31.59 ± 13.47
MAP [mmHg]	115.4 ± 19.92
Brachial SBP [mmHg]	141.41 ± 25.89
Brachial DBP [mmHg]	97.77 ± 16.59
Brachial PP [mmHg]	43.64 ± 16.61
PP amplification	1.41 ± 0.10
TPR [mmHg.s/ml]	1.13 ± 0.23
Total arterial compliance [ml/mmHg]	1.97 ± 0.69
Aortic diameter [mm]	28.57 ± 1.95
Height [cm]	175.00 ± 25.00

DBP, diastolic blood pressure; MAP, mean arterial pressure; PP, pulse pressure; SBP, systolic blood pressure; SD, standard deviation; t_ad_, ending time point of isovolumic contraction; t_ed_, early time point of isovolumic contraction; t_es_, end-systolic time point; TPR, total peripheral resistance;

PP amplification = Brachial PP/Aortic PP.

### Comparison Between Estimated Elastance and Real Elastance

**FIGURE 3:**
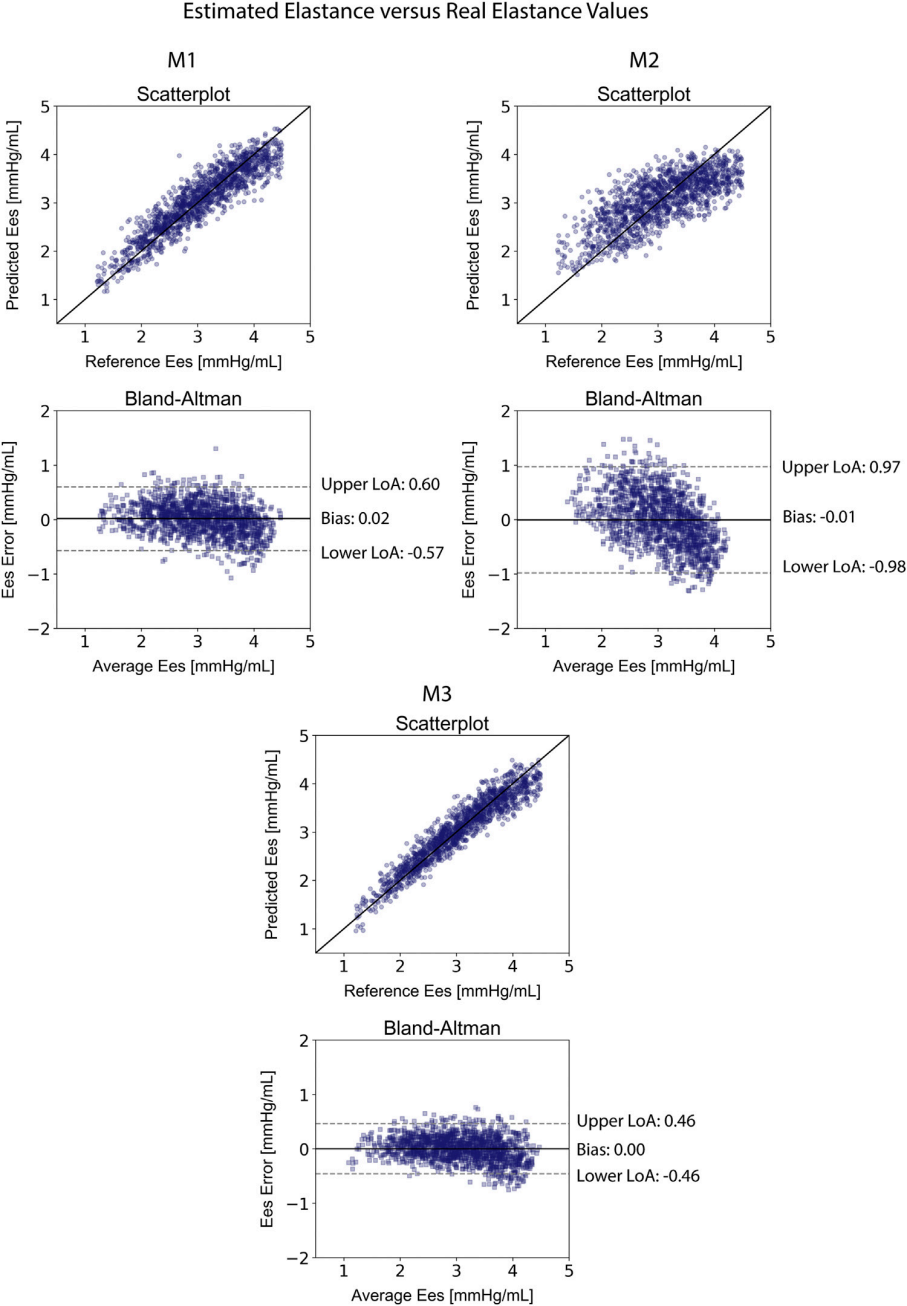
Comparison of the estimated E_es_ values with the reference E_es_ for the three predictive models M1, M2, and M3. Scatterplots between the values of E_es_ derived from the models and the real E_es_. Solid line represents equality. Bland-Altman plot for estimated E_es_ and real E_es_ for Extreme Gradient Boosting. Limits of agreement (LoA), within which 95% of errors are expected to lie, are defined by the two horizontal dashed lines.

Table 4 displays the statistical comparisons between the noninvasive E_es_ estimates and the reference E_es_. The Bland-Altman plot shows that the estimated E_es_ had low bias. The limits of agreement (LoA) between the estimated and reference E_es_ (within which 95% of errors are expected to lie) were found to be [−0.57, 0.60] mmHg/ml. The scatterplot and the Bland-Altman plots of the estimated E_es_ against the real E_es_ are presented in Figure 3. Finally, standard error of estimate (SEE) was reported to be 0.15 mmHg/ml. The absolute difference between the noninvasive E_es_ estimates and the real E_es_ values was reported to be lower than 0.5 mmHg/ml in 91% of the total cases for XGB. At large, the regressor performed adequately towards the accurate prediction of E_es_.

**TABLE 4:** Regression statistics between model-predicted and reference data.

Model	Slope	Intercept	r	*p*-value	RMSE	nRMSE (%)	MAE
XGB_Ees_ M1	0.82	0.57 mmHg/ml	0.92	<0.0001	0.30 mmHg/ml	9.15	0.24 mmHg/ml
XGB_Ees_ M2	0.52	1.45 mmHg/ml	0.74	<0.0001	0.50 mmHg/ml	15.26	0.41 mmHg/ml
XGB_Ees_ M3	0.88	0.38 mmHg/ml	0.95	<0.0001	0.24 mmHg/ml	7.32	0.19 mmHg/ml
XGB_Vd_ M1	0.00	22.55 ml	<0.1	0.79	14.14 ml	25.79	11.92 ml
XGB_Vd_ M2	0.00	22.58 ml	<0.1	0.79	14.14 ml	25.79	11.91 ml
XGB_Vd_ M3	0.86	3.28 ml	0.93	<0.0001	5.00 ml	9.12	3.62 ml

MAE, mean absolute error; nRMSE, normalized RMSE; r, Pearson’s correlation coefficient; RMSE, root mean square error; SD, standard deviation; XGB, Extreme Gradient Boosting.

Two-sided *p*-value for a hypothesis test whose null hypothesis is that the slope is zero, using Wald Test with t-distribution of the test statistic.

M1 uses brachial systolic blood pressure (brSBP), brachial diastolic blood pressure (brDBP), heart rate (HR), pre-ejection period (PEP), ejection time (ET), early time point of isovolumic contraction (t_ed_), ending time point of isovolumic contraction (t_ad_), and end-systolic time point (t_es_); M2 uses brSBP, brDBP, HR, PEP, and ET; M3 uses all features from M1 as well as stroke volume and ejection fraction.

**FIGURE 4:**
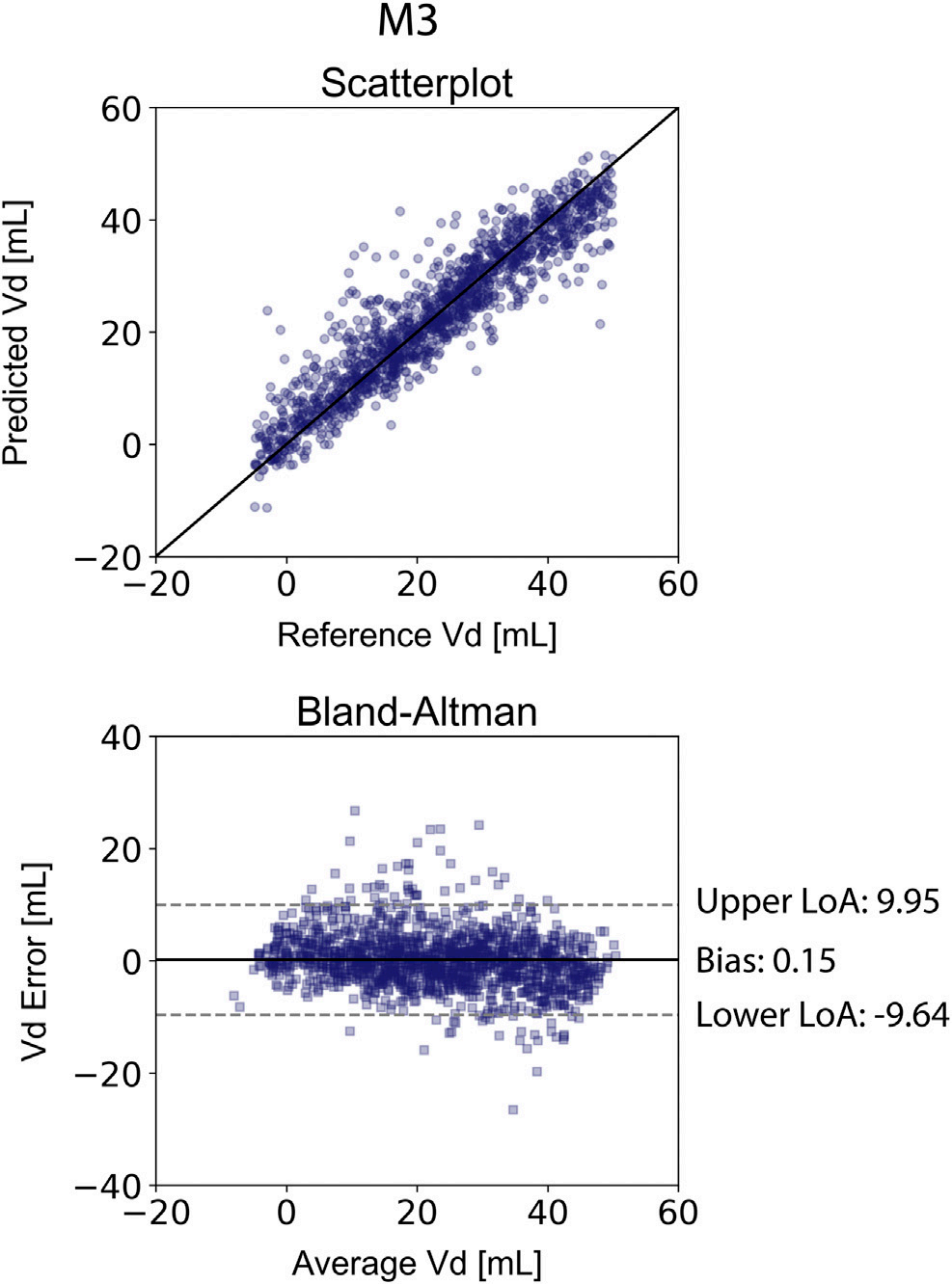
Comparison of the estimated V_d_ values with the reference V_d_ for the XGB_Vd_ M3 model. Scatterplots between the values of V_d_ derived from the model and the real V_d_. Solid line represents equality. Bland-Altman plot for estimated E_es_ and real E_es_ for Extreme Gradient Boosting. Limits of agreement (LoA), within which 95% of errors are expected to lie, are defined by the two horizontal dashed lines.

The results for the V_d_ estimation are also reported in Table 4. For the XGB_Vd_ M1 and XGB_Vd_ M2 models, no agreement was achieved between the predictions and the reference data (r < 0.1). Inclusion of the SV and EF led to improved accuracy, achieving a nRMSE equal to 9.12% and a correlation of 0.93. Figure 4 illustrates the scatterplot and the Bland-Altman plot for the predicted and real V_d_ values only for the best-performing model (XGB_Vd_ M3).

Table 5 presents the average permutation importances of the input features, sorted in descending order for predicting E_es_. Following the concept of permutation, t_ed_, t_es_, and PEP yielded the highest increase in the prediction error on test data (increase in RMSE was equal or more than 0.46 mmHg/ml). The XGB-based feature importances are also given in Table 5. PEP had a critical contribution (0.440) followed by t_ad_ and t_es_ with 0.186 and 0.107, respectively.

**TABLE 5:** Feature importances for the prediction of E_es_.

Feature	Permutation importance (mmHg/ml) *mean ± SD*	Importance score by XGB
t_ed_	1.583 ± 0.019	0.099
t_es_	1.408 ± 0.020	0.107
PEP	0.458 ± 0.011	0.440
brDBP	0.109 ± 0.004	0.073
brSBP	0.086 ± 0.003	0.030
ET	0.056 ± 0.003	0.015
HR	0.024 ± 0.002	0.050
t_ad_	0.005 ± 0.001	0.186

brDBP, brachial diastolic blood pressure; brSBP, brachial systolic blood pressure; ET, ejection time; HR, heart rate; PEP, pre-ejection period; t_ad_: ending time point of isovolumic contraction: t_ed_: early time point of isovolumic contraction; t_es_: end-systolic time point; XGB: extreme gradient boosting.

### Sensitivity to Measurement Errors

When the systolic time intervals, i.e., PEP and ET, were randomly overestimated or underestimated, the performance of the model gradually deteriorated. Concretely, corruption of the data with random noise gave a rise to the error between the predictions and reference values. The performance of the model for the different levels of noise is presented in Table 6. Standard deviation of the RMSE values at the noise levels was ±0.11 mmHg/ml. At the level of maximal noise (±30%), RMSE reached the value of 0.55 mmHg/ml, while the Pearson’s correlation coefficient substantially decreased at 0.68. The estimated E_es_ values were considerably influenced by noise corruption.

**TABLE 6:** Regression statistics between model-predicted E_es_ and reference E_es_ when artificial noise is considered.

Model	Slope	Intercept	r	*p*-value	RMSE	nRMSE (%)	MAE
XGB_Ees_ M1 (noise-free)	0.82	0.57 mmHg/ml	0.92	<0.0001	0.30 mmHg/ml	9.15	0.24 mmHg/ml
XGB_Ees_ M1 (± 10% noise in STIs)	0.72	0.87 mmHg/ml	0.84	<0.0001	0.41 mmHg/ml	12.51	0.33 mmHg/ml
XGB_Ees_ M1 (± 20% noise in STIs)	0.59	1.26 mmHg/ml	0.74	<0.0001	0.50 mmHg/ml	15.26	0.40 mmHg/ml
XGB_Ees_ M1 (± 30% noise in STIs)	0.54	1.40 mmHg/ml	0.68	<0.0001	0.55 mmHg/ml	16.78	0.44 mmHg/ml
XGB_Ees_ M1 (± 10% noise in BP)	0.83	0.53 mmHg/ml	0.92	<0.0001	0.30 mmHg/ml	9.15	0.24 mmHg/ml
XGB_Ees_ M1 (± 20% noise in BP)	0.81	0.58 mmHg/ml	0.91	<0.0001	0.31 mmHg/ml	9.46	0.24 mmHg/ml
XGB_Ees_ M1 (± 30% noise in BP)	0.81	0.57 mmHg/ml	0.91	<0.0001	0.32 mmHg/ml	9.76	0.25 mmHg/ml

BP, blood pressure; E_es_, end-systolic elastance; MAE, mean absolute error; nRMSE, normalized RMSE; r, Pearson’s correlation coefficient; RMSE, root mean square error; SD, standard deviation; STI, systolic time intervals; XGB, Extreme Gradient Boosting.

Two-sided *p*-value for a hypothesis test whose null hypothesis is that the slope is zero, using Wald Test with t-distribution of the test statistic.

M1 uses brachial systolic blood pressure (brSBP), brachial diastolic blood pressure (brDBP), heart rate (HR), pre-ejection period (PEP), ejection time (ET), early time point of isovolumic contraction (t_ed_), ending time point of isovolumic contraction (t_ad_), and end-systolic time point (t_es_); M2 uses brSBP, brDBP, HR, PEP, and ET; M3 uses all features from M1 as well as stroke volume and ejection fraction.

Errors in brachial blood pressure measurements impacted to a lesser extent the estimation of E_es_. With increasing the magnitude of the introduced noise, we did not notice a pronounced variation in the RMSE after the noise level of 20%, namely RMSEs varied by ±0.01 mmHg/ml. When the noise level was ±30%, RMSE found to be equal to 0.32 (r = 0.91) for the XGB model. Overall, cardiac elastance values were minimally affected.

## Discussion

In the present study, we found that end-systolic elastance could be estimated noninvasively from arm cuff pressure and systolic time intervals following a Machine Learning approach. We developed and tested our method using synthetic data from a previously validated in-silico model of cardiovascular dynamics. The study population corresponded to an extensive range of cardiac and arterial systemic conditions. The regression results showed that cuff pressure in conjunction with systolic time intervals (STIs) achieved a low test error and can capture the LV E_es_ value with sufficient accuracy. The present work is in line with previous efforts towards the noninvasive estimation of E_es_ using easily obtained single-beat noninvasive measurements.

In our previous study (Bikia et al., 2020), we demonstrated that the noninvasive estimation of E_es_ can be achieved when arm cuff pressure, carotid-to-femoral pulse wave velocity (cfPWV), and EF are used as inputs to a regressor. Conventionally, EF is often used to assess LV systolic function and can be measured using different cardiac imaging technics, including magnetic resonance imaging (MRI), the Simpson’s method, speckle tracking strains, etc. However, these imaging modalities are tedious and require a highly trained technician. To facilitate the assessment of cardiac performance, several studies have focused on the use of STIs which can be conveniently obtained via Pulse Doppler echocardiography (Weissler et al., 1968; Weissler et al., 1981; Reant et al., 2010). Motivated by this concept, we chose to reformulate the regression pipeline for the estimation of E_es_ and replace EF with simple systolic timing parameters. A strong argument reinforcing our methodology arrives from the fact that interpretation of EF is limited when preload and afterload are not known (Krayenbühl et al., 1968).

The XGB model achieved high accuracy in the estimated E_es_ with r = 0.92. In 91% of the total cases, the average difference between the noninvasive E_es_ and the reference E_es_ was reported to be lower than 0.50 mmHg/ml. Given that, for a normal heart, E_es_ lies within the ranges of [1.50–3.50] mmHg/ml, while for dilated hearts and hypertrophied hearts is near 1.00 mmHg/ml and 4.00 mmHg/ml, respectively (Chen et al., 1998; Chen et al., 2001), such an error should allow for reasonably accurate assessment of systolic function in normal and pathological hearts.

Furthermore, based on the learning curve (Figure 2), the training error was reported to be low, and, hence, the training data are fitted well by the estimated model (low bias). The small gap between the two curves indicated a low variance. The learning curve well predicted a low RMSE close to 0.29 mmHg/ml for the training data size equal to or larger than 4,000. Based on this learning curve, we can deduce that our particular predictive model needs a training dataset of 4,000 to reach an error of 0.29 mmHg/ml. These findings could be utilized as a starting reference point for future studies that develop similar estimators.

The individual time points, i.e., t_ed_ (early time point of isovolumic contraction), t_ad_ (ending time point of isovolumic contraction), and t_es_ (end-systolic time point), were incorporated in the input to enhance the performance of the model. In the spirit of completeness, we further investigated the change in the accuracy of the predictive model when the latter time points were not considered as input features. In that case, the XGB model predicted E_es_ achieving an RMSE equal to 0.50 mmHg/ml and a correlation coefficient of 0.74. The feature importances were re-ranked as follows: PEP: 0.555, brDBP: 0.154, brSBP: 0.103, HR: 0.101, and ET: 0.087. Given the deterioration in the accuracy, we chose to keep the aforementioned time points (given that they are available when PEP and ET are measured) in the input vector in order to maximize our model’s performance.

In order to further evaluate the robustness of our models, we quantified the effect that measurement errors might have on the E_es_ estimates. Concretely, we performed the regression analysis while introducing artificial noise to the STIs and the brachial pressure recordings. An erroneous measurement of the STIs appeared to have a greater impact on the E_es_ estimation compared to an error in the brachial blood pressure features. Overall, the sensitivity analysis on errors in the input features demonstrated that estimated E_es_ values were considerably affected by random errors in the systolic timing features (namely, t_ed_, t_ad_, t_es_, PEP, and ET). In contrast, the overall regression performance was altered only slightly when random noise corrupted brSBP and brDBP without significantly affecting the accuracy of the estimated E_es_ values. This can be further explained if we consider the permutation feature importances for our model; the timing intervals and, in particular, t_ed_ and t_es_ held the first places in the ranking (RMSE would increase at least by 1.4 mmHg/ml after permutating one of those two features).

Based on the permutation feature importances, the time points t_ed_ and t_es_ were the most significant contributors to the precise estimation of E_es_. If permutation of a feature leads to a predictive model with insufficient prediction capacity (high errors), then the information provided by this feature is significant and the corresponding feature is considered as important. The threshold for an error to indicate poor prediction is dependent on the problem under consideration. In the present study, the error threshold for a precise estimation was set to be lower than 0.50 mmHg/ml, and, therefore, all the features with permutation importances leading to errors higher than the threshold were considered as largely important. The discrepancies in the features’ ranking between the two approaches for calculating the importance level can be explained by the fact that the one is based on the training process, while the other one relies on the predictions on the testing dataset. Moreover, the feature importance method by XGB favors features that have high cardinality. In our dataset, all PEP values were unique for all the 4,645 data instances, and this might encourage the algorithm to consider it as the most important feature. It is recommended that interpretation of the importances is done in a combinational manner, so that a more complete overview is provided using different insights and aspects. Yet, PEP had a critical contribution using both concepts. The high correlation between PEP and LV function has been also demonstrated by previous studies (Reant et al., 2010). Finally, the important contribution of brDBP (4th higher increase in error) can be explained by the fact that brDBP is strongly related to the mean arterial blood pressure, which indicates the pressure against which the heart pumps.

### Clinical Application of the Proposed Method

Systolic time intervals can be easily and precisely measured in the clinical practice and may be used for detecting alterations in LV systolic function (Boudoulas, 1990). The correlation between these STIs measurements and conventional LV function parameters has been emphasized in numerous previous studies (Reant et al., 2010) paving the way to further explore the potential in using more complicated nonlinear Machine Learning approaches.

**FIGURE 5:**
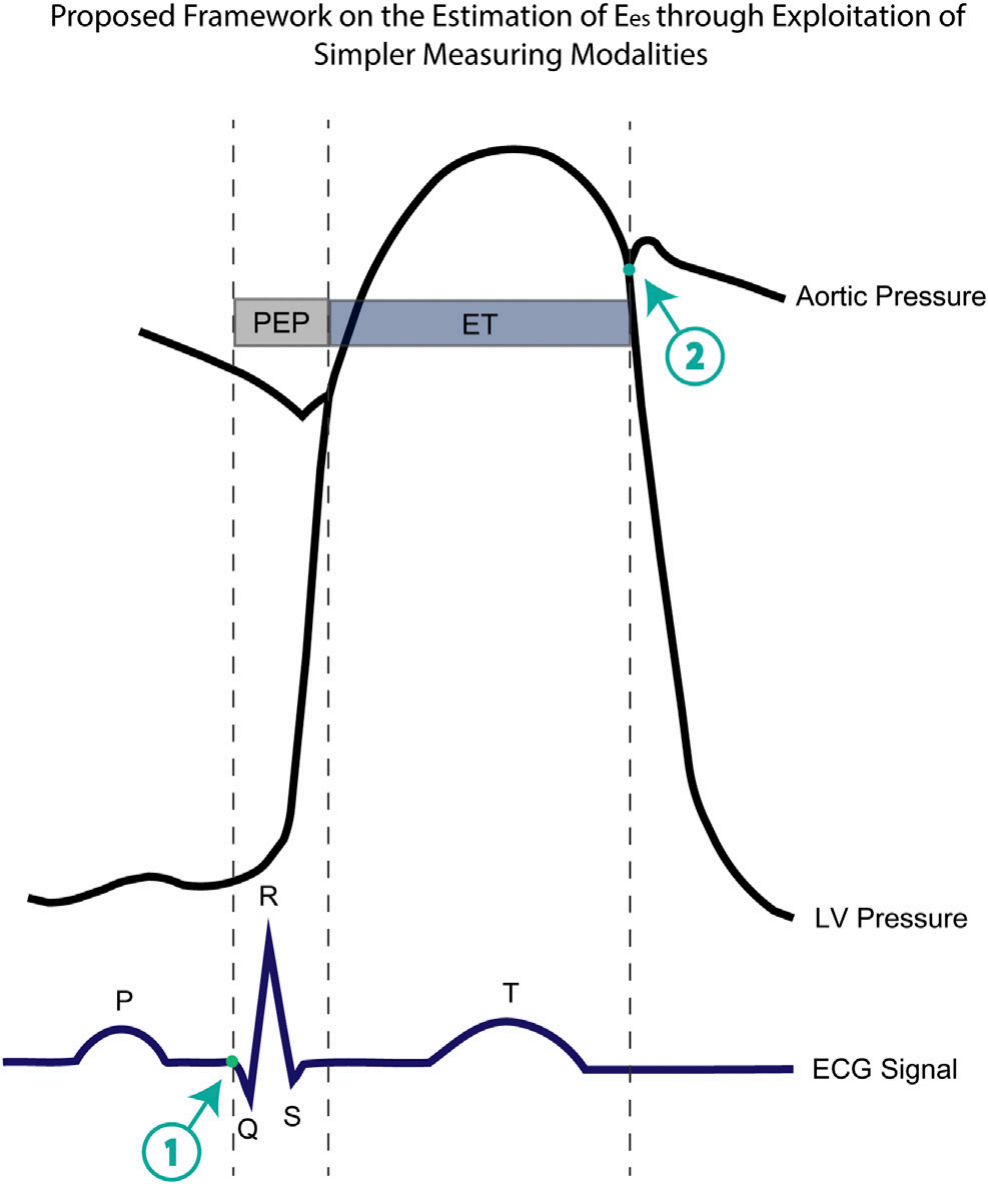
Representation of the aortic pressure waveform, the left ventricular pressure, the ECG electrocardiogram including the timing components of pre-ejection period (PEP), ejection time (ET), and the newly introduced Q-aoClos interval. The Q-aoClos interval is the time period from the initial trace of Q-wave (point 1) (as measured via ECG) until the closure of the aortic valve (point 2) (as recorded via a phonographic device).

From a wider perspective, the incorporation of STIs values as features to approximate E_es_ has been a promising research direction. Several researchers have demonstrated the ability in acquiring these STIs measurements from more simplified modalities including electrocardiography (ECG), phonocardiography (PCG), or seismocardiography (SCG) (Dehkordi et al., 2019; Chung et al., 2020). Such methods provided unobtrusive detection of cardiac time intervals and offer the potential to be integrated into wearable devices. Interestingly, PEP and ET could be very easily obtained using ECG and a precise electronic stethoscope. More concretely, the initiation of the PEP interval is placed at the initial point of the Q-wave (point 1, Figure 5). In addition, an electronic stethoscope able to capture the time intervals in the scale of milliseconds would allow us to determine the moment of the aortic valve closure (point 2, Figure 5). Now, if we set a new time interval which is the sum of PEP and ET (Q-aoClos interval, Figure 5), we can measure the exact duration of the latter using ECG and stethoscope alone. The ECG signal could indicate the initiation of Q-wave, while phonocardiography would allow us to detect the closure of the aortic valve. To test this hypothesis, we performed the regression analysis using as inputs only the arm cuff pressure, the Q-aoClos interval, namely, the summation of PEP and ET, the time point at the beginning of Q-wave (time 1), and the time point at the closure of the aortic valve (point 2). Our results indicated that E_es_ could be effectively estimated achieving an nRMSE and Pearson’s correlation coefficient equal to 10.37% and 0.89, respectively, wheareas LoA were ±0.67 mmHg/ml and bias was zero. In that case, the selected hyperparameters were *learning_rate* = 0.05, *max_depth* = 3, *n_estimators* = 1,250. This finding creates a rather promising proof-of-evidence towards the noninvasive estimation of E_es_ reducing the complexity and the cost of the technique for acquiring the necessary measurements. The proposed methodological concept could be easily integrated in a medical device such as a smart stethoscope.

### Prior Work on the E_es_ Estimation

Several methods have been proposed for the E_es_ estimation using noninvasive single-beat measurements. First, Chen *et al.* (Chen et al., 2001) proposed a simple equation for estimating E_es_ from arm cuff pressure, SV, and EF. Their proposed method incorporates an estimated normalized ventricular elastance at arterial end-diastole which was derived from regression on previously recorded studies. The authors achieved accurate estimations with differences between estimated and real values equal to 0.43 ± 0.50 mmHg/ml and a high correlation of 0.91. Here, we decided, however, to simplify our method by replacing the measurements of the stroke volume and ejection fraction with the more accurately obtained pre-ejection period and ejection time intervals. In addition, the calculation of EF as assessed by echocardiography can be rather sensitive to errors and derived approximately. Removal of EF from our calculation can reduce the error imposed by such an approximation.

Moreover, Shishido *et al.* (Shishido et al., 2000) suggested the estimation of E_es_ from pressure values, systolic time intervals, and stroke volume. Their analysis relies on the approximation of the time-varying elastance curve by two linear functions corresponding to the isovolumic contraction phase and the ejection phase. The slope ratio of these functions is calculated and used for estimating E_es_ by the employment of a simple equation. Their model provided reliable predictions of E_es_ in anesthetized dogs with r = 0.93 and SEE = 2.10 mmHg/ml. In accordance with our findings, this methodology evidences the utility of systolic time intervals on the estimation of E_es_. A limitation of their study pertains to the fact that the authors developed their model using the same population which was used for the model’s testing rather than an independent group.

Recently, Pagoulatou *et al.* (Pagoulatou et al., 2021) proposed and validated a novel method for noninvasively estimating E_es_ based on sphygmomanometric pressure measurements and standard echocardiographic examination, comprising the measurement of aortic flow and ejection fraction. Their method is based on the adjustment of the aforementioned model of the cardiovascular system to patient-specific standards and subsequently allows for the derivation of E_es_ and V_d_ via an inverse model-fitting approach. Invasive validation of their technique on 19 patients yielded accurate estimates of $E_{es}$ [r = 0.89, nRMSE = 9%, bias = −0.13 mmHg/ml with limits of agreement (−0.9, 0.6) mmHg/ml], while it was demonstrated that the method is robust to measurement noise.

### Limitations

This study has potential limitations that need to be acknowledged. The major limitation of the present study is the use of synthetic data and not real *in vivo* recordings. Nevertheless, synthetic data can sufficiently simulate the content of the real clinical measurements, while they allow for controlling the distribution of rare but relevant conditions or events. In addition, the in-silico model that was used for the data generation has been thoroughly validated against *in vivo* data and provides realistic representations of the physiological signals. Another limitation pertains to the fact that PEP and ET used for the training/testing scheme were extracted from the elastance curve, albeit this framework has been designed to use only echocardiographic measures. This approach was selected due to the lack of ECG information, given that cardiac electrical events are not yet included in our in-silico model. Sensitivity analysis was performed in order to examine the model’s performance with respect to over- and underestimation of these two features. Furthermore, our proposed method does not provide the entire ESPVR, given that the inputs do not provide adequate information to predict V_d_. However, we observed that when the SV and EF were included in the input vector, our method is able to estimate V_d_ with an nRMSE = 9.12% and r = 0.93. Finally, the current database was created using the mathematical model of a healthy individual free of pathology. Hence, implementation of the method is limited in cases of aortic valve stenosis, regurgitation, or other valve pathologies, where the relationship between the peripheral pressure and the STIs is modified. Further investigation towards this direction will be performed in our future studies.

## Conclusion

This study provided evidence that accurate estimates of E_es_ could be yielded from pressure data and contractility-related timing parameters using a data-driven approach. Based on our findings, we conclude that data-driven approaches might be valuable for estimating E_es_. The STIs appeared to be a promising source of information for assessing E_es_ and their usefulness should be emphasized. At large, our results were found to be in good agreement with the actual E_es_ values over an extensive range of LV contractility values and loading conditions. The proposed methodological concept could be easily transferred to the bedside and potentially facilitate the clinical use of E_es_ for monitoring the contractile state of the heart in the real-life setting.

## Data Availability

The data generated and analyzed in the present study are available from the corresponding author on a reasonable request.
